# A brief review about melatonin, a pineal hormone

**DOI:** 10.20945/2359-3997000000066

**Published:** 2018-08-01

**Authors:** Fernanda Gaspar do Amaral, José Cipolla-Neto

**Affiliations:** 1 Universidade Federal de São Paulo Universidade Federal de São Paulo Departamento de Fisiologia São Paulo SP Brasil Departamento de Fisiologia, Universidade Federal de São Paulo (Unifesp), São Paulo, SP Brasil; 2 Universidade de São Paulo Universidade de São Paulo Instituto de Ciências Biomédicas Departamento de Fisiologia e Biofísica São Paulo SP Brasil Departamento de Fisiologia e Biofísica, Instituto de Ciências Biomédicas, Universidade de São Paulo (USP), São Paulo, SP, Brasil

**Keywords:** Melatonin, pineal, hypermelatoninemia, hypomelatoninemia

## Abstract

Melatonin is a ubiquitous molecule in nature, being locally synthesized in several cells and tissues, besides being a hormone that is centrally produced in the pineal gland of vertebrates, particularly in mammals. Its pineal synthesis is timed by the suprachiasmatic nucleus, that is synchronized to the light-dark cycle via the retinohypothalamic tract, placing melatonin synthesis at night, provided its dark. This unique trait turns melatonin into an internal synchronizer that adequately times the organism's physiology to the daily and seasonal demands. Besides being amphiphilic, melatonin presents specific mechanisms and ways of action devoted to its role as a time-giving agent, being widely spread in the organism. The present review aims to focus on melatonin as a pineal hormone with specific mechanisms and ways of action, besides presenting the clinical syndromes related to its synthesis and/or function disruptions.

## MELATONIN

**Figure 1:**
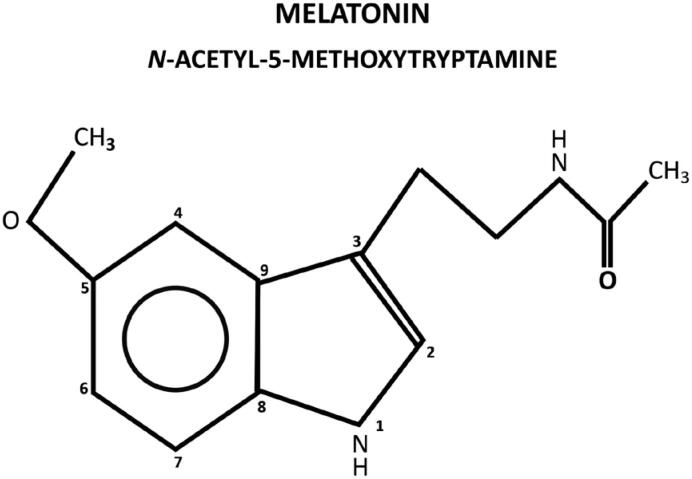
Melatonin molecule (232,2 molecular weight).

Melatonin, N-acetyl-5-methoxytryptamine, is a ubiquitous molecule in nature, being found in almost all living organisms. It is an indolamine present in any compartment of the organism for its amphiphilic characteristics of diffusion (Figure 1). In vertebrates, mammals in particular, in addition of local production in several tissues, melatonin is centrally produced by the pineal gland and directly released in the blood, acting as a hormone. The pineal gland is an unpaired epithalamic neuroendocrine gland originating from the roof of the third ventricle containing, in mammals, melatonin-producing cells called pinealocytes, in addition to astrocytes and other cell types (1).

**Figure 2:**
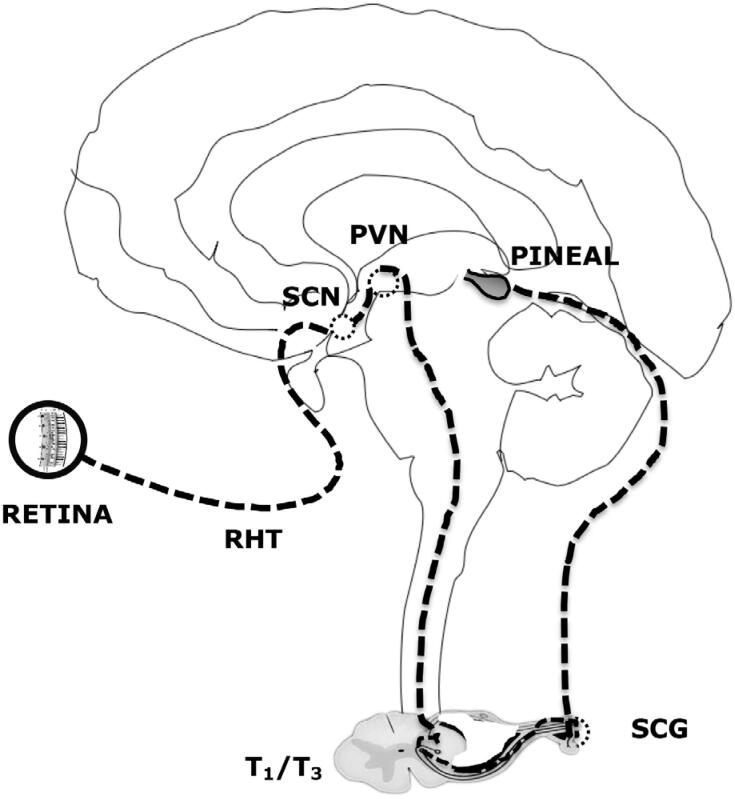
Neural control of pineal melatonin synthesis. RHT: retinohypothalamic tract. SCN: suprachiasmatic nucleus. PVH: paraventricular nucleus. SCG: superior cervical ganglion.

Melatonin synthesis by the pinealocytes in the pineal gland is under the control of a neural system originating in the hypothalamic paraventricular nuclei, projecting directly and indirectly to the preganglionic sympathetic neurons of the first thoracic segments of the spinal cord. Following, through a projection of the postganglionary sympathetic neuron of the superior cervical ganglia, nerve fibers forming the conary nerves reach the pineal gland (Figure 2).

Norepinephrine released by the sympathetic terminals interacts with the classical beta and alpha noradrenergic receptors in the membrane of pinealocytes and activates cAMP-PKA-CREB and PLC-Ca++-PKC pathways to trigger melatonin synthesis (2).

**Figure 3:**
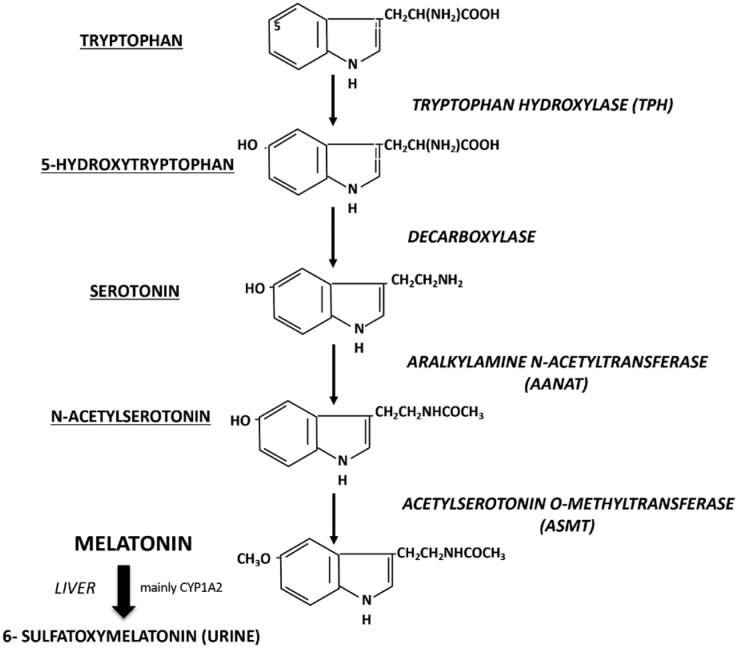
Melatonin synthesis pathway and hepatic metabolization. The enzymes are written in italic and the derived molecules are underlined.

Melatonin synthesis initiates with tryptophan that, under the action of tryptophan hydroxylase, is transformed in 5-hydroxytryptophan that, in turn, is converted to serotonin, which is acetylated, by arylalkylamine N-acetyltransferase (AANAT) to N-acetylserotonin (NAS) that is converted to melatonin by acetylserotonin O-methyltransferase (ASMT) former called hydroxy-indole-O-methyltransferase (HIOMT). The three enzymes above are under the control of neural and endocrine systems that regulate time, duration and amount of produced melatonin (3) (Figure 3).

The major control is exerted by the circadian timing system, mainly the hypothalamic suprachiasmatic nuclei, that times melatonin synthesis so that it is daily produced in synchrony to the light/dark cycle, being tightly restricted to the night, provided it is dark. Light stimulus (mainly in the blue range) activates melanopsin breakdown in retinal photoreceptive ganglion cells that project, via the retinohypothalamic pathway, to the hypothalamus, inhibiting melatonin synthesis (4).

Due to its amphiphilic nature, melatonin is not stored inside the pinealocytes, being released as it is synthetized. The pineal gland is profusely vascularized and its attachment, dorsal and posterior, to the third ventricle wall allows melatonin to be released into the cerebrospinal fluid of the central nervous system during the night, as well as into the blood stream. In the blood, melatonin is usually bound to albumin, metabolized to 6-hydroxymelatonin by cytochrome P450 isoforms (mainly CYP1A2) and conjugated to 6-sulfatoxymelatonin in the liver, for the subsequent urinary excretion. 6-sulfatoxymelatonin production perfectly reflects the plasma levels of melatonin, so its urinary measurement is a less intrusive method to evaluate the pineal function and melatonin production (Figure 3). In the central nervous system melatonin is degraded to N-acetyl-N2-formyl-5-methoxykynuramine (AFMK) that is deformylated to N-acetyl-5-methoxykynuramine (AMK) (5).

**Figure 4:**
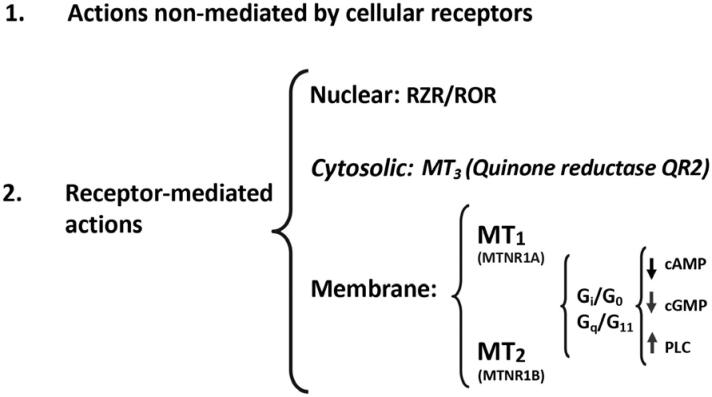
Melatonin mechanisms of action. Classical receptor mediated and non-mediated pathways and the involvement of nuclear, cytosolic and membrane receptors.

Melatonin, as an ancient chemical messenger, developed several pleiotropic mechanisms of action (6). First, there are mechanisms that are not mediated by cellular receptors and involve the direct interaction of melatonin and other molecules, as its antioxidant action. Melatonin is one of the most powerful natural antioxidants, not only by directly chelating oxygen and nitrogen reactive species, but also by mobilizing the intracellular antioxidant enzymatic system. Second, as any other hormone, melatonin acts through specific cellular receptors. Membrane melatonin receptors, in mammals, are of two types, MT1 (MTNR1A, in humans) and MT2 (MTNR1B in humans). These membrane melatonin receptors are heterotrimeric Gi/ Go and Gq/11 protein-coupled receptors that interact with downstream messengers such as adenylyl cyclase, phospholipase A2 and phospholipase C, generally decreasing cAMP and cGMP production and/or increasing diacylglycerol and IP3 formation. MT1 and MT2 receptors are found in almost all peripheral tissues, as well as in the central nervous system. In addition to acting through membrane receptors, melatonin might interact with ROR/RZR (retinoid orphan receptors/ retinoid Z receptors) nuclear receptors (7) (Figure 4).

As mentioned before, melatonin hormonal pineal production is restricted to the night and a light signal, mainly with the characteristics of day-light (predominance of the blue range), blocks melatonin secretion. This complex and very well-organized neural system control is the product of natural selection that turned the melatonin nocturnal profile into the internal representative of the environmental night. In addition, and as a consequence, the duration of melatonin nocturnal secretion episode follows the duration of the night as it varies along the year. Long winter nights determine long plasma melatonin duration episodes and the reverse occurs following the short nights of summer time.

Melatonin synthesis circadian rhythm synchronized to day/night cycle, restricted to the night and to its duration, converts melatonin to the internal representative of the daily and seasonal photoperiod. As a consequence of being a representative of the environmental photoperiod, melatonin has, in some way, to control the organism physiology during the 24 hours of the day and all through the seasons of the year. To do that, melatonin, using the classical hormonal mechanisms of action, developed several especial ways of action (8).

**Figure 5:**
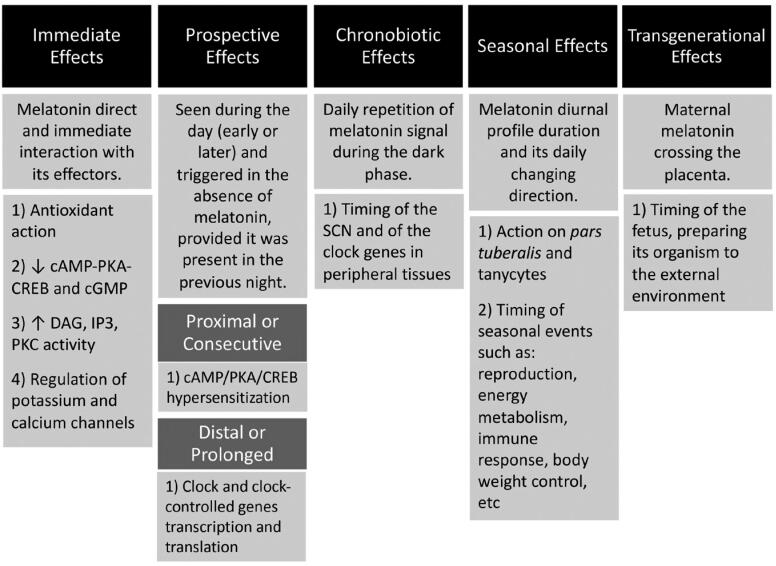
Melatonin ways of action. The upper boxes represent the different ways of action. The second-line boxes explain how melatonin causes the correspondent effects. The third-line boxes show examples of the correspondent effects. Note that the Prospective effects are further classified in Proximal ou consecutive and Distal or prolonged effects.

As any other hormone, melatonin acts through the classical way in the sense that its effects are seen as a direct and immediate consequence of its interaction with molecular effectors. These are called *immediate effects* (Figure 5). Depending on the molecular effectors and the involved mechanisms of action, such are the possible effects: antioxidant action; reduction of cAMP-PKA-CREB and cGMP; increased DAG, IP3, PKC activity; regulation of potassium and calcium channels; etc. As it would be expected for any hormone, the effects are dependent on the target tissue and the correspondent intracellular melatonin signaling pathway.

However, in addition to this classical and expected hormonal way of action, melatonin developed another one that is not seen during the night when melatonin is being produced and released but, instead, it is only seen during the day, being triggered in the absence of plasma melatonin, provided it was present in the immediate previous night. These are called *prospective effects* and they are of two types (Figure 5). The first one, called *proximal or consecutive,* is seen right at the beginning of the morning, immediately after the cessation of melatonin production, when it is maximal and may extend to several hours. One example is the cAMP/ PKA/CREB pathway super or hypersensitization that is seen in several peripheral and central systems (9). That is to say that in consequence of the nocturnal sustained and prolonged adenylyl cyclase inhibition induced by melatonin through its G_i_-protein coupled receptors, this signaling pathway shows, following the cessation of the inhibitory signal, an increased and potentiated response to any agonists that activates adenylyl cyclase through any G_s_-protein coupled receptors. Depending on the system (suprachiasmatic nucleus, pancreatic beta cells, Leydig cells, *pars tuberalis,* etc.) such is the magnitude and the duration of the potentiating prospective consecutive melatonin effect (10-13). That is to say that melatonin, in spite of being produced only during the night, determines effects that are best seen during the day, when melatonin is not present anymore, and in this case, especially in the morning. The second type of melatonin prospective effects are called *distal or prolonged effects.* These are dependent on the action of melatonin controlling the transcription and/or translation of the well-known clock genes (CG) and the clock-controlled genes (CCG) (14-20). The clock genes are part of a complex molecular machinery that includes a cycle of transcription and translation of several genes and the resulting proteins might either reinforce the process or inhibit it, so that the cycle has a duration of approximately 24 hours (21). These proteins can also control, throughout the 24-hour cycle, the transcription and translation of other genes, called clock-controlled genes, that are responsible for almost all cell functions. That is to say that melatonin, through its prospective prolonged effects, controlling the cycling of the CG and CCG, is able to control cell and tissue function all over the 24-hours of the day, even being only produced and released during the night.

Mediated by these immediate and prospective effects, melatonin developed others ways of action. One of them depends on the circadian characteristic of the melatonin signal and on the contrast between nocturnal and diurnal values of circulating melatonin. The precise daily repetition of melatonin signal and its perfect relation to the dark phase of the day turns melatonin into the internal synchronizer of circadian rhythms. This shapes the so-called *chronobiotic effect* of melatonin (Figure 5). Melatonin is one of the most powerful synchronizers of human circadian rhythms and is used in clinics to adjust circadian rhythmicity in cases like phase-delayed sleep onset disorder, jet lag, etc. (2225) The chronobiotic effect of melatonin depends on its action at several levels of the circadian timing system, including the suprachiasmatic nucleus and the clock genes located in peripheral tissues like, adipose tissue, muscle, pancreatic beta cells, reproductive organs, etc.

Another important synchronizing effect of melatonin is related to the seasonal rhythms. It is its *seasonal effect* (26-28) (Figure 5). The duration of melatonin diurnal profile and, most importantly, the direction of the daily change of its duration (towards increasing or decreasing nights resulting in increasing or decreasing duration of melatonin production) that is dependent on the typical relative duration of the day and night along the year, turns melatonin into the most powerful synchronizer of seasonal rhythms, being fundamental for the organism to anticipate and to adapt to the evolving environmental change along the year. The seasonal synchronizing effect of melatonin is mediated by its action on the pituitary *pars tuberalis.* From there, the signal is transduced to the hypothalamus, mediated by special glial cells called tanycytes, and to the distal hypophysis through several other mediators. In consequence of this functional system, melatonin is able to control seasonal events like reproduction, energy metabolism, immune response and thermogenesis, growth, body weight control, etc. (29-33).

Finally, another melatonin way of action called *transgenerational effect* should be mentioned (Figure 5). In mammals, in humans in particular, pineal melatonin production increases as the gestation progress (34). Maternal melatonin freely crosses the placenta and reaches the fetus circulation, being its only source of melatonin (35-37). Considering this, several of the effects of melatonin that can be seen in the maternal organism are seen in the fetus, particularly, the chronobiotic and seasonal effects (38-40). Maternal melatonin is responsible for the circadian timing and priming of the fetus organism. Similarly, the seasonal timing is transferred from the mother to the fetus, preparing its neuroendocrine system to the future environment to be dealt with (41,42). In addition, maternal melatonin is necessary for the adequate neurodevelopment of the fetus (43).

## MELATONIN PHYSIOLOGY, CLINICAL SYNDROMES AND THERAPEUTICS

Melatonin, due to its phylogenetic history, its pleiotropic mechanisms of action and its unique ways of action, as described above, in addition of being delivered to the blood stream and directly in the CNS, is able to regulate several, if not all, the physiological and neural functions. Among them, circadian and seasonal timing of organism; sleep and wakefulness cycle; endocrine functions, as energy metabolism, glycemic control, blood lipid profile and reproduction, gestation and fetal development and programming; cardiovascular system; immune system; neural development, neural protection and neuroplasticity, etc. A detailed discussion of this subject can be seen in Cipolla-Neto and Amaral, 2018 (8).

As any other hormone, from the clinical point of view, melatonin hormonal dysfunction can be classified as hypo *(hypomelatoninemia)* or hyper *(hypermelatoninemia)* production by the pineal gland.

Hypomelatoninemia is defined by decreased melatonin nocturnal peak value or total production when compared to what is expected for the age- and sex-paired population. This syndrome can be classified as *primary*, dependent on factors that directly affect the pineal gland and/or its innervation, and *secondary,* developed as a consequence of a primary event, such as a systemic disease (e.g. hyperglycemia) or environmental factor (e.g light at night) (44-50).

Hypermelatoninemia is defined as hyperproduction of pineal melatonin, usually associated to other diseases like hypogonadotrophic hypogonadism, anorexia nervosa, polycystic ovarian syndrome, Rabson-Mendenhall syndrome and spontaneous hypothermia hyperhidrosis (51-54).

A third syndrome associated to pineal melatonin dysfunction is due to what can be called *inappropriate melatonin receptor-mediated response.* This melatonin-receptor dysfunction is usually a consequence of melatonin receptors genetic variations (e.g. single nucleotide polymorphisms) and affect either MT1 or MT2 receptors (55,56).

Finally, in addition to these classical hormonal dysfunctions and due to melatonin specific characteristics of production and ways of action, it is possible to define another syndrome associated to the time displacement of the nocturnal melatonin production causing a phase-displacement of its plasma profile that is called *melatonin circadian displacement syndrome.* The result is a misalignment of the organism to the circadian timing domain, causing sleep/wake, metabolic and cardiovascular disturbances, among other symptoms. This syndrome is usually associated to the Smith-Magenis disease, the phase-delayed sleep-wake disorder, and as a consequence of indoors illumination during the evening/night, among others (57,58).

Melatonin pharmacokinetics will depend on the way of administration (oral, fast and/or slow-release, intravenous, nasal spray, anal suppository, skin patches or cream, etc.) and on the individual absorption and hepatic metabolization rates (dependent on the activity of cytochrome P450 complex, mainly CYP1A2). Each of these aspects might vary depending on age and sex. Usually, in a young/middle-aged human patient, pharmacokinetic studies show that plasma concentration reaches the peak at approximately 45 minutes after orally administered melatonin, resulting in a low bioavailability due to the first pass liver metabolism (59).

Melatonin administration should always be done during the evening/night, mimicking the physiological production. The moment of administration during the evening/night will depend on the desired effect that will be determined by the well-known melatonin phase-response curve. That is to say that depending on the moment of administration, melatonin is able to act on the circadian clock resulting in phase-advance, phasedelay or even no phase-displacement of the circadian rhythms. If administered in the late afternoon/ beginning of the evening, melatonin phase-advances the circadian rhythms (as is the case for the treatment of the sleep-wake phase-delay disorder); if administered in the end of the night/early morning, melatonin would phase-delay the circadian clock; if administered during the evening, beginning around 1 hour before the usual bedtime and extending to 2 to 4 hours afterwards, melatonin does not phase displace the circadian rhythms, regulating the circadian clock pace (this is the time of choice if melatonin is being replaced, as in the pinealectomized or elder patients).

It should be said that, considering the above discussed melatonin ways of action, one should always avoid its chronic administration during the day.

The dosage is always a concern and will depend on the desired effect. If replacement therapy is the goal, oral melatonin in the range of 0.1 to 0.5 mg usually generates a plasma concentration varying from 100 to 500 pg/mL, that is 1 to 5 times the expected physiological concentration at the nocturnal peak in young people. In addition, 0.5 to 1.0 mg is usually used for proper circadian timing, as in jetlag for example. Melatonin-responsive sleep disorders are usually treated with 1.0 to 5.0 mg oral melatonin. However, one should always keep in mind that the dosage must be individually adjusted by the evaluation of the evolution of the symptoms and the occurrence of adverse effects as diurnal somnolence or nocturnal nightmares.

## CONCLUSIONS

Nowadays, any quick search in PubMed/NCBI shows that there are about 33,000 papers about melatonin or pineal and about 1,200 new articles per year, in addition to a dedicated journal (Journal of Pineal Research) showing the 2017 impact factor of 11.613 (6th out of 143 journals in the area of Endocrinology & Metabolism). In spite of this, melatonin hormonal action is scarcely targeted in the classical Physiology or Endocrinology books. In Williams’ textbook of Endocrinology, for example, the pineal gland was included for a long time in the circumventricular organs section and is, nowadays, located in a separate scanty part just after it. Jameson & De Groot devoted, for a long time, a full chapter to the pineal gland that is mostly dedicated to pineal tumors. Melatonin in all its complexity is barely reported. In addition, in several medical schools all over the world, pineal gland and/or melatonin is never systematically taught. So, it is about time to change this picture, considering melatonin as a hormone decisively important to the mammalian and human physiology and pathophysiology.

Concerning the clinical aspects of melatonin dysfunction some observations should be additionally done. The most common mistake that we find in clinical endocrinology when dealing with melatonin is to expect, as far as hypo or hyper production is concerned, exactly the same that we would be expected to occur with the classical glands syndromes: immediate effects and immediate health repercussions. It is not the case with pineal and melatonin as it is a timedomain acting hormone that organizes physiology and behavior in the circadian and seasonal time. Its absence induces an unhealthy state whose clinical pictures is not so tinted as is expressed in other glands but is still there and will be reflected in the *long-term health status* rather then immediately. For example, a subtle alteration in the sleep organization as little as shorter 30 minutes sleep episode every day will not be even perceived by the patient and the physician. However, it will determine, in the medium/long term, several critical alterations in metabolism (insulin resistance, overweight, etc.), cardiovascular system (hypertension), loss of performance, GIT events, reproduction/sexual repercussions, etc. (60). It should be considered, still, that the sleep/wake cycle is only one among several others affected systems in melatonin-deficient adults and children. Extend this to several of the circadian aspects of physiology and the clinical picture becomes much more serious, resulting in systemic repercussion reaching every other aspect of human physiology and behavior, jeopardizing its health and quality of life and even longevity.
